# RT-DETR-DCEA: A Lightweight Citrus Defective Fruit Detection Algorithm for Complex Orchard Environments

**DOI:** 10.3390/plants15132077

**Published:** 2026-07-03

**Authors:** Jihui Qiao, Yuchen Sun, Binyuan Zhong, Lun Wang, Siyu Li, Hang Liu, Youqing Chen, Tong Li

**Affiliations:** 1 College of Mechanical and Electrical Engineering, Yunnan Agricultural University, Kunming 650201, China; 2 The Key Laboratory for Crop Production and Smart Agriculture of Yunnan Province, Yunnan Agricultural University, Kunming 650201, China; 3 School of Big Data, Yunnan Agricultural University, Kunming 650201, China

**Keywords:** RT-DETR, defective citrus fruit, lightweight detection, dynamic convolution, multi-scale feature fusion, adaptive sparse attention

## Abstract

Given the issues in natural orchard environments, such as large-scale variations of defective citrus fruits, weak texture boundaries, strong illumination changes, branch and leaf occlusion, and significant background interference, this paper constructs a lightweight detection model, RT-DETR-DCEA, based on RT-DETR-R18. This model is improved through four aspects: “fine-grained defective feature extraction—multi-scale feature fusion—up-sampling detail recovery—global feature interaction for noise suppression”. First, a Dynamic Hybrid Convolution Module (DIMB) is introduced into the backbone network, drawing on the ideas of Inception-style multi-branch depthwise convolution and MetaFormer residual mixing. It extracts local textures of various forms through square convolution, horizontal strip convolution, and vertical strip convolution, and utilizes dynamic branch weights to enhance the model’s adaptability to irregular defects such as lesions, mildew, and external damage. Second, a Content-Guided Attention Feature Fusion Network (CGAFN) is designed in the neck network, which achieves adaptive fusion of low-level detail features and high-level semantic features through channel attention, spatial attention, and pixel-level fusion weights. Next, a lightweight upsampling enhancement module called EUCB-SC is constructed, which introduces channel rearrangement and Shift spatial offset into the efficient upsampling convolutional structure to enhance the local spatial interaction capability of upsampled features with low parameter overhead. Finally, adaptive sparse self-attention is introduced into the AIFI module to form AIFI-ASSA, which suppresses irrelevant background interactions through a sparse attention branch and retains necessary contextual information through a dense attention branch. The experimental results demonstrate that on a dataset containing four categories of citrus images—healthy, diseased, moldy, and severely externally damaged—RT-DETR-DCEA achieves 92.1% Precision, 86.1% Recall, and 91.8% mAP@50, with a parameter count of 1.477 × 10^7^ and an inference speed of 81 FPS. Compared with the original RT-DETR-R18 and various YOLO series models, this method strikes a favorable balance among detection accuracy, recall capability, and model lightweightness. This paper also discusses limitations such as data scale, ratio of private data, single training result, and insufficient validation on edge devices, providing a basis for subsequent cross-regional data validation and real-world deployment testing.

## 1. Introduction

Citrus is a globally important economic fruit, and its appearance quality and defect status directly affect commodity grade, postharvest storage, and circulation safety [1]. During growth, harvesting, and transportation, fruits may develop defects such as lesions, mold, collision-induced dents, peel tears, and insect damage. In early stages, these defects often manifest as small areas, low contrast, and weak boundary textures. Reliance on manual inspection or sorting is not only inefficient but also susceptible to variations in experience, fatigue, and changes in ambient lighting [2]. With the advancement of smart orchard and automated sorting technologies, establishing a lightweight visual model capable of stably identifying multiple categories of citrus defects in complex natural scenes has clear engineering value.

In recent years, the YOLO series, DETR series, and improved models have been widely used for agricultural object detection [3]. YOLO-type models offer advantages such as fast inference speed and mature engineering deployment, but they are still prone to missed detections or false positives on small objects, occluded objects, and backgrounds with similar colors [4,5,6,7,8,9,10,11,12]. DETR-type models reduce manual design processes such as anchor boxes and non-maximum suppression through set prediction and the Transformer architecture, and possess strong global modeling capabilities [13,14,15,16,17,18,19]; among them, RT-DETR further improves the real-time performance of end-to-end detection and has been gradually applied to tasks such as fruit ripeness recognition, crop disease detection, and agricultural object perception in complex scenes. Existing studies have shown that improved RT-DETR can increase mAP and reduce model size or computational complexity in fruit detection. However, most works still focus on single targets, single environments, or obvious differences in target appearance. Their adaptability to scenes with multiple categories of defects, weak-texture lesions, and complex orchard backgrounds still needs to be strengthened [20,21,22,23,24,25,26,27].

For the task in this paper, the original RT-DETR-R18 still has three deficiencies. First, conventional convolutions or fixed-receptive-field convolutions have limited adaptability to variations in defect morphology, making it difficult to simultaneously capture multi-morphological features such as spot-like mildew, flaky lesions, and directional lacerations. Second, if cross-scale fusion in the neck network relies solely on addition, concatenation, or fixed weights, high-level semantics are prone to overshadowing low-level details, causing the edge information of small defects to decay during multi-layer propagation. Third, standard dense self-attention computes relationships between all spatial positions in complex orchard backgrounds, which may introduce noisy interactions from irrelevant regions such as foliage, shadows, highlights, and healthy fruit skin textures. The aforementioned problems collectively limit the detection performance of the model for small-scale, multi-category, and weak-boundary defects under lightweight constraints.

To address these issues, this paper constructs RT-DETR-DCEA. The main objectives of this paper include: (1) improving detection accuracy for small-scale and weak-boundary defects; (2) reducing model parameters while maintaining real-time inference capability; (3) enhancing robustness against occlusion, strong light, and similar-texture interference in complex orchard backgrounds; (4) verifying the independent contributions and combined effects of each module on performance improvement through ablation experiments. The main contributions of this paper are as follows.

1. A Dynamic Inception-style Mixed Convolution Block (DIMB) is proposed. By introducing input-adaptive branch weights into Inception-style multi-pattern depthwise convolutions, DIMB enhances the backbone network’s ability to represent irregular defect textures.
2. A Content-Guided Attention Feature Fusion Network (CGAFN) is designed to dynamically assign fusion weights to low-level detail features and high-level semantic features according to the input content, thereby improving the quality of multi-scale defect feature fusion.
3. A lightweight upsampling enhancement module, EUCB-SC, is constructed. By introducing channel shuffle and Shift-based spatial displacement into the efficient upsampling convolution of EUCB, EUCB-SC enhances local spatial information interaction with a low parameter overhead.
4. Introducing ASSA into the Attention-based Intra-scale Feature Interaction (AIFI) stage of RT-DETR forms AIFI-ASSA, which suppresses interference from low-relevance backgrounds while preserving essential global context.

## 2. Materials and Methods

### 2.1. Citrus Data Acquisition

**Figure 1:**
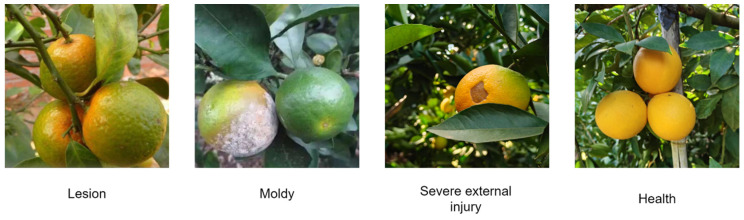
Images of citrus fruits with different defects and healthy citrus fruits.

The experimental study area was located at 24.25° N, 99.06° E, supported by the Yunnan Key Laboratory of Crop Production and Smart Agriculture, and combined with the Chu Orange production base in Longling County, Baoshan City, Yunnan Province, China. This study focused on the Chu orange cultivar from Yunnan Chushi Agriculture Co., Ltd., supplemented with a large amount of citrus data collected from other regions to improve the recognition accuracy and generalization capability of the model under different regional and environmental conditions. The images were manually collected using an Intel RealSense D455 camera manufactured by Intel Corporation, Santa Clara, CA, USA. Examples of the collected images are shown in Figure 1.

Traditional citrus defect classification generally includes healthy fruit and three common defect categories: diseased fruit, moldy fruit, and fruit with severe external damage. Samples with extensive lesions exhibited clear gradient characteristics in lesion coverage. The affected area ranged from approximately one-third of the peel surface to nearly the entire fruit, with color gradually changing from light brown to dark brown. Some samples were also accompanied by peel shrinkage or gum exudation. Moldy samples were mainly characterized by fungal colonies, with mold spots appearing in white, green, or black. Their morphological patterns included punctate aggregation, patchy spreading, and annular diffusion, and some samples showed visible details of hyphae penetrating peel cracks. Samples with severe external damage included typical defects such as impact-induced depressions, peel tearing, and insect-bored holes. The damaged regions commonly showed tissue rupture, browning caused by juice loss, or discolored patches resulting from external contamination. By constructing a sample set containing typical morphological variations, the ability of deep learning models to recognize the spatial distribution characteristics of lesions can be enhanced, thereby improving the detection and recognition performance for various defective fruits in complex field environments. This study focused on citrus fruits with lesions, mold infection, and severe external damage, with healthy citrus fruits used as controls. Each type of citrus fruit was annotated according to the above method, as shown in Figure 1.

### 2.2. Overall Architecture of RT-DETR-DCEA

**Figure 2:**
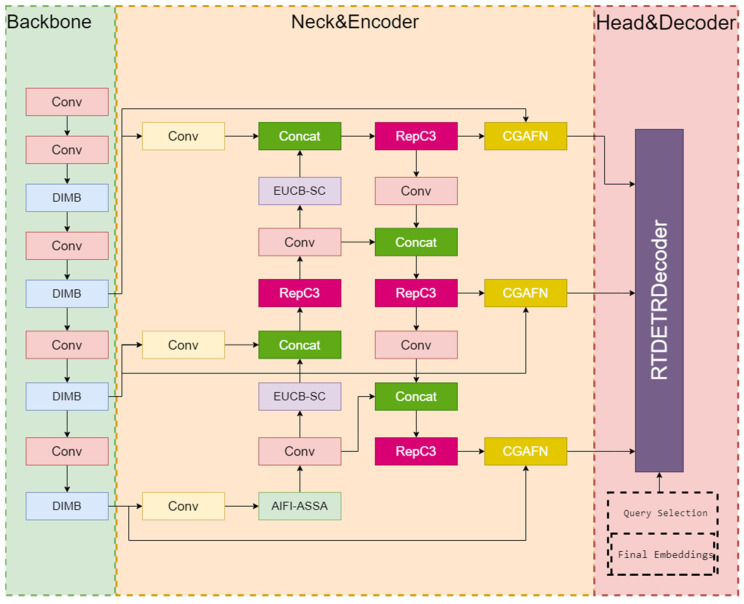
The improved RT-DETR network architecture diagram.

This study adopts RT-DETR-R18 as the baseline model and introduces improvements at four key stages: feature extraction, cross-scale fusion, upsampling enhancement, and global feature interaction. The overall framework is illustrated in Figure 2. Specifically, DIMB is embedded into the backbone network to strengthen the local representation of defect textures with diverse morphological patterns. CGAFN is used to replace or enhance the original multi-scale fusion structure in the neck, allowing the fusion weights of features from different levels to be adaptively determined according to the input content. EUCB-SC is applied to the upsampling path to improve the recovery of local details in upsampled features. AIFI-ASSA is further introduced to optimize intra-scale feature interaction within AIFI, thereby reducing the interference of low-relevance regions in complex backgrounds during defect feature aggregation.

It should be noted that the contributions of the modules in this study mainly lie in their structural adaptation and integrated optimization for citrus defect fruit detection, rather than in reinterpreting existing modules as entirely independent new theories. To avoid overstatement, the relationships between the proposed modules and the relevant reference works are explicitly described in Section 2, and their practical contributions to the present task are further validated through ablation experiments.

#### 2.2.1. RT-DETR Baseline Model

RT-DETR is an end-to-end real-time object detection framework that extracts multi-scale features using a convolutional backbone network and performs set prediction through an efficient hybrid encoder and a Transformer decoder [28,29,30,31,32,33]. Compared with conventional anchor-based detectors, RT-DETR reduces the influence of anchor design and NMS post-processing on the detection pipeline, achieving a favorable balance between detection speed and accuracy [34,35,36,37,38,39]. In RT-DETR, the AIFI module is mainly used to model global interactions among high-level features, whereas the CCFM module is responsible for multi-scale cross-layer feature fusion. In this study, RT-DETR-R18 is selected as the lightweight baseline model because its parameter size and inference speed are more suitable for agricultural detection tasks on mobile or edge devices.

#### 2.2.2. DIMB Dynamic Mixed Convolution Module

**Figure 3:**
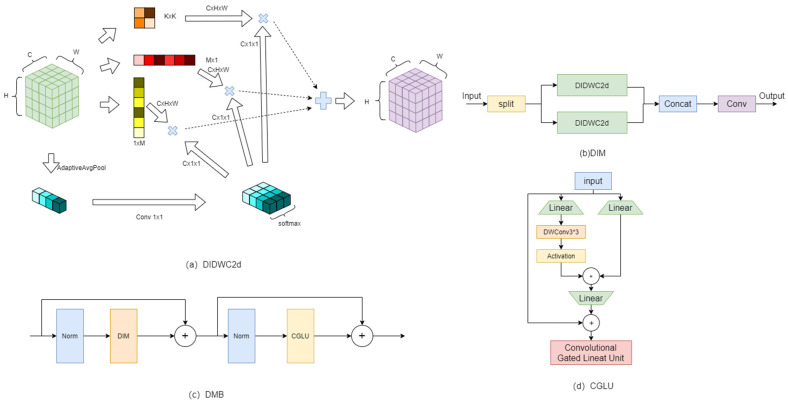
Network architecture diagrams of DIDWC2d, DIM, and DMB.

Citrus defects exhibit substantial morphological variations. Lesions may appear as spot-like, patch-like, or irregularly spreading regions; cracks and mechanical injuries may present directional edges; and moldy areas are often accompanied by fine-grained texture variations. A single fixed convolution kernel is therefore insufficient to simultaneously capture local spots, elongated textures, and directional edges. To address this issue, inspired by the multi-branch depthwise convolution design in InceptionNeXt and the general structural paradigm of “normalization–feature mixing–channel mapping–residual connection” in MetaFormer [40,41], this study constructs a Dynamic Inception Mixer Block (DIMB) to enhance the backbone network’s representation capability for citrus defect features with diverse morphologies. The structure of DIMB is shown in Figure 3.

The core component of DIMB is DynamicInceptionDWConv2d. This structure consists of three parallel depthwise convolution branches, namely a square depthwise convolution branch, a horizontal strip depthwise convolution branch, and a vertical strip depthwise convolution branch. Specifically, the square convolution branch is used to extract local textures and spot-like defect features; the horizontal strip convolution branch is designed to capture horizontally extended cracks, edges, or texture structures; and the vertical strip convolution branch is employed to extract vertically elongated damage edges and structural information. Let the input feature be defined as:

(1) $$X\in R^{C\times H\times W},$$

The three depthwise convolution branches can then be formulated as:

(2) $$F_{s}s={DWConv}_{k\times k}(X),$$

(3) $$F_{h}={DWConv}_{1\times m}(X),$$

(4) $$F_{v}v={DWConv}_{m\times 1}(X),$$

where $F_{s}$, $F_{h}$ and $F_{v}$ denote the output features of the square convolution branch, horizontal strip convolution branch, and vertical strip convolution branch, respectively. DWConv represents the depthwise convolution operation, while k×k, 1×m and m×1 indicate convolution kernels with different shapes.

Different from static multi-branch fusion strategies, DIMB further introduces a dynamic branch-weighting mechanism for convolutional branches. Specifically, global average pooling is first applied to extract global contextual information from the input feature. Then, a 1×1 convolution is used to generate three sets of branch weights, which are normalized by the Softmax function:

(5) $$[\alpha_{s},\alpha_{h},\alpha_{v}]=Softmax({Conv}_{1\times 1}(GAP(X))),$$

where $\alpha_{s}$, $\alpha_{h}$, and $\alpha_{v}$ denote the dynamic weights corresponding to the three convolution branches, respectively, and GAP(·) represents the global average pooling operation.

The outputs of the three branches are fused through dynamic weighted aggregation:

(6) $$Y=\alpha_{s}F_{s}+\alpha_{h}F_{h}+\alpha_{v}F_{v},$$

This mechanism enables the model to adaptively adjust the contributions of different convolution branches according to the morphological variations of defect regions in the input image. When defects appear as local spots or small-area lesions, the square convolution branch can provide stronger local texture responses. In contrast, when defects appear as cracks, scratches, or directional edges, the horizontal and vertical strip convolution branches can more effectively capture directional structural information.

In DynamicIncMixerBlock, DynamicInceptionMixer serves as the spatial token mixer to achieve multi-morphological feature mixing along the spatial dimension. A convolutional GLU or 1×1 feed-forward mapping is used as the channel-mixing unit to enhance the nonlinear representation capability in the channel dimension. Meanwhile, this module incorporates residual connections, Layer Scale, and DropPath to improve training stability and reduce the risk of overfitting. The overall computational process can be formulated as:

(7) $$X^{\prime }=X+DropPath(\lambda_{1}\cdot Mixer(Norm(X))),$$

(8) $$Y=X^{\prime }+DropPath(\lambda_{2}\cdot MLP(Norm(X^{\prime }))),$$

where $\lambda_{1}\;$ and $\lambda_{2}$ denote the learnable Layer Scale parameters, Mixer(·) represents the DynamicInceptionMixer, and MLP(·) denotes the channel-mapping unit.

It should be emphasized that the innovation scope of DIMB does not lie in re-proposing Inception-style depthwise convolution itself. Rather, its contribution lies in combining Inception-style multi-morphology depthwise convolution with an input-adaptive branch-weighting mechanism and adapting this design to the RT-DETR backbone network. This enables the model to better extract fine-grained textures, directional edges, and irregular lesion features in citrus defect fruit detection. In this way, DIMB preserves the efficiency of multi-branch depthwise convolution while improving the model’s dynamic perception capability for different defect morphologies.

#### 2.2.3. CGAFN Content-Guided Attention Feature Fusion Module

In complex orchard scenes, features at different levels contain markedly different types of information. Low-level features usually preserve rich edge, color, and texture details, making them sensitive to small lesions, mold boundaries, and mechanical damage edges. In contrast, high-level features contain stronger category semantics and contextual information, which helps suppress interference caused by branch and leaf occlusion, shadows, specular highlights, and complex backgrounds. If multi-scale features are directly fused by simple addition or concatenation, low-level details may be overwhelmed by high-level semantic information, thereby weakening the responses of small-scale defects, weak-texture lesions, and edge-damage regions.

**Figure 4:**
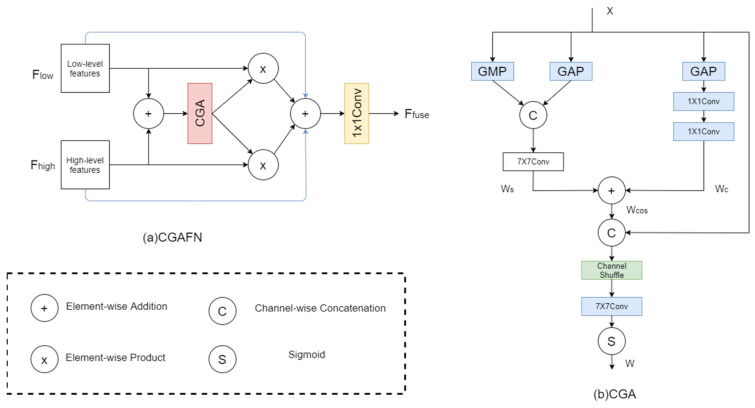
CGAFN Network Architecture Diagram.

To address the above issue, inspired by the content-guided attention mechanism and CGA-based mixup fusion strategy in DEA-Net [42,43], this study constructs a Content-Guided Attention Feature Network (CGAFN) to achieve content-guided cross-scale feature fusion in the detection neck, as shown in Figure 4. The core idea of this module is to adaptively adjust the contributions of two input features during fusion according to their channel semantics, spatial distributions, and pixel-level responses, rather than using a simple fusion strategy with fixed weights. Let the two features to be fused be denoted as:

(9) $$F_{x}\in R^{C\times H\times W},$$

(10) $$F_{y}\in R^{C\times H\times W},$$

where $F_{x}$ and $F_{y}$ denote features from different scales or different pathways, respectively.

The two feature branches are first fused as follows:

(11) $$F_{init}=F_{x}+F_{y},$$

Channel attention and spatial attention are then applied to the initially fused feature $F_{init}$ to extract importance information along the channel and spatial dimensions, respectively:

(12) $$A_{c}=CA(F_{init}),$$

(13) $$A_{s}=SA(F_{init}),$$

where CA(·) denotes the channel attention operation, which is used to model the importance of different feature channels, while SA(·) denotes the spatial attention operation, which is employed to locate the response intensity of defect-related regions along the spatial dimension.

After summing the two attention maps, a coarse-grained attention guidance map is obtained:

(14) $$A=A_{c}+A_{s},$$

Based on this, a pixel attention module is further introduced to jointly model the initially fused feature and the coarse-grained attention map, generating fine-grained fusion weights:

(15) $$W=\sigma (PA(F_{init},A)),$$

where PA(·) denotes the pixel attention operation, σ(·) represents the Sigmoid activation function, and W is the final generated adaptive fusion weight. This weight can dynamically regulate the contribution ratio of the two input features across both channel and spatial positions.

The output feature of CGAFN can be formulated as:

(16) $$F_{out}={Conv}_{1\times 1}(F_{init}+W⊙F_{x}+(1-W)⊙F_{y}),$$

where ⊙ denotes element-wise multiplication, and ${Conv}_{1\times 1}$ is used for channel rearrangement and fused-feature projection. This fusion strategy not only preserves the initial information from the two feature branches, but also adaptively enhances the more discriminative feature components through the weight W. When defect boundaries are weak or texture details are prominent, the model can increase the contribution of low-level detailed features. In contrast, when the background is complex or the target is occluded, the model can rely more on high-level semantic features to reduce background interference.

Unlike conventional spatial attention, the fusion weights in CGAFN are not determined by a single spatial weight map alone, but are jointly generated by channel attention, spatial attention, and pixel attention. This design enables the module to address three key questions simultaneously: first, which channels are more important for defect recognition; second, which spatial regions are more likely to contain defect-related information; and third, how the contributions of the two feature branches should be allocated during pixel-level fusion. Therefore, CGAFN can regulate the multi-scale feature fusion process in a more fine-grained manner, thereby improving the representation capability of the neck network for small-scale lesions, mold textures, and mechanical damage boundaries.

In citrus defect fruit detection, defect regions are usually characterized by small areas, blurred boundaries, weak color variations, and strong background interference. Through its content-guided adaptive fusion mechanism, CGAFN helps enhance the feature responses of defect-related regions while reducing interference from irrelevant backgrounds, such as branches and leaves, shadows, specular highlights, and healthy peel textures. It should be emphasized that this study does not claim to propose the CGA mechanism for the first time. Instead, it transfers and adapts the existing content-guided fusion strategy to the neck network of RT-DETR to address the insufficient fusion of multi-scale defect features in agricultural scenarios.

#### 2.2.4. EUCB-SC Efficient Upsampling and Shifted Channel Mixing Module

In the detection neck, upsampling is mainly used to restore the spatial resolution of high-level feature maps, enabling cross-scale fusion with low-level detailed features. The upsampling operations in the original neck structure usually rely on nearest-neighbor interpolation or bilinear interpolation. Although these methods can restore the feature map size, they do not inherently provide feature enhancement capability. For citrus defect fruit detection, small lesions, mold boundaries, and mechanical damage regions often exhibit small scales, weak edges, and fragmented textures. If further feature refinement is lacking after upsampling, the responses of defect boundaries may become insufficient, or local details may be lost.

**Figure 5:**
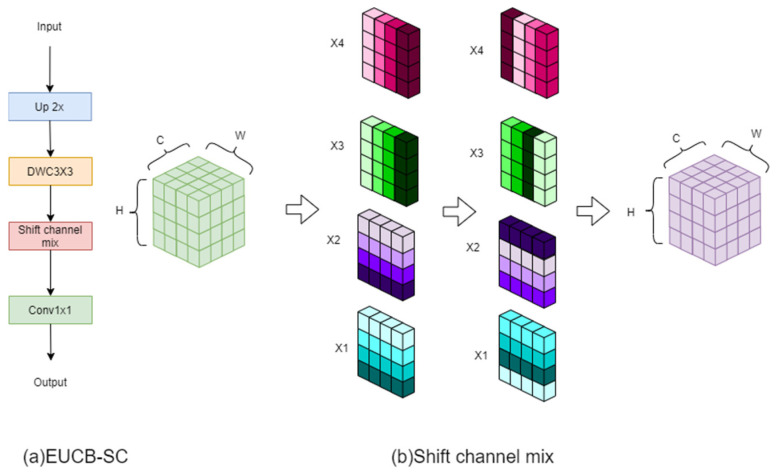
Network structure diagram of EUCB-SC module.

To improve feature recovery during the upsampling stage, this study draws on the design concept of the Efficient Up-Convolution Block (EUCB) in EMCAD [44] and further introduces the Shift Channel Mix operation to construct the EUCB-SC module, as shown in Figure 5. The original EUCB achieves efficient feature recovery through upsampling, depthwise convolution, and 1×1 pointwise convolution, thereby enhancing local spatial representation after upsampling with low computational cost. Considering that depthwise convolution mainly performs spatial modeling within individual channels and has limited capability for inter-channel information interaction, this study further incorporates channel rearrangement and cyclic shift operations to enhance spatial information flow across different channels. Let the input feature be denoted as:

(17) $$X\in R^{C\times H\times W},$$

EUCB-SC first upsamples the input feature by a factor of 2 and then enhances the local spatial representation of the upsampled feature through depthwise convolution:

(18) $$F_{u}=DWC(U_{p}(X)),$$

where Up(·) denotes the upsampling operation, DWC(·) represents the depthwise convolution operation, and $F_{u}$ denotes the feature obtained after upsampling and depthwise convolution.

The feature is then rearranged along the channel dimension and divided into four sub-feature groups:

(19) $$F_{u}=[F_{1},F_{2},F_{3},F_{4}],$$

where $F_{1}$, $F_{2}$, $F_{3}$ and $F_{4}$ denote the four channel subgroups, respectively.

To enhance information interaction among neighboring spatial positions, the four feature groups are cyclically shifted forward or backward along the height and width dimensions, respectively:

(20) $$F_{1}^{\prime }=Roll(F_{1},+s,H),$$

(21) $${F_{2}}^{\prime }=Roll(F_{2},-s,H),$$

(22) $${F_{3}}^{\prime }=Roll\left(F_{3},+s,W\right),$$

(23) $${F_{4}}^{\prime }=Roll(F_{4},-s,W),$$

where s denotes the shift step size; H and W represent the height and width dimensions, respectively; and Roll(·) denotes the cyclic shift operation.

The four shifted feature groups are then concatenated again along the channel dimension, followed by a 1×1 convolution for channel fusion and feature projection:

(24) $$F_{sc}=Concat(F_{1}^{\prime },{F_{2}}^{\prime },{F_{3}}^{\prime },{F_{4}}^{\prime }),$$

(25) $$Y={Conv}_{1\times 1}(F_{sc}),$$

where Concat(·) denotes the channel-wise concatenation operation, and Y represents the output feature of EUCB-SC.

The Shift Channel Mix operation does not introduce additional learnable parameters. Instead, it enables feature channels to acquire information from neighboring spatial positions through spatial rearrangement. Compared with standard depthwise convolution, this operation can expand the local information interaction range with extremely low computational overhead. Compared with ordinary upsampling, EUCB-SC not only restores the spatial resolution but also further enhances the local representation capability of the upsampled features. By combining channel rearrangement with 1×1 convolution, this module can compensate for the insufficient channel interaction of depthwise convolution, allowing information from different channels to be further fused.

For citrus defect fruit detection, EUCB-SC helps improve the upsampled representation quality of small defect boundaries, weak-texture lesions, and local mechanical damage regions. Particularly under conditions of fruit occlusion, uneven illumination, and strong interference from background branches and leaves, this module can enhance local detail information with low parameter and computational overhead, thereby providing a more stable feature basis for subsequent cross-scale feature fusion. It should be noted that the contribution of EUCB-SC does not lie in re-proposing upsampling or the shift operation itself. Instead, it combines an efficient up-convolution block with a parameter-free shifted channel mixing mechanism and adapts it to the neck network of RT-DETR, thereby meeting the dual requirements of detail preservation and lightweight inference in citrus defect detection.

#### 2.2.5. AIFI-ASSA Adaptive Sparse Feature Interaction Module

The AIFI module in RT-DETR is mainly used to enhance global interaction within single-scale features. Through the self-attention mechanism, AIFI can model long-range dependencies among different spatial positions, thereby improving the representation capability of high-level semantic features. However, in citrus defect fruit detection, image backgrounds are usually complex and often contain defect-irrelevant information, such as branch and leaf occlusion, shadows, specular highlights, and natural peel textures. If dense self-attention is directly used to compute the correlations among all spatial positions, low-relevance background regions will also participate in feature aggregation. This may introduce noisy interactions and weaken the responses of small lesions, moldy regions, and weak-texture defects.

**Figure 6:**
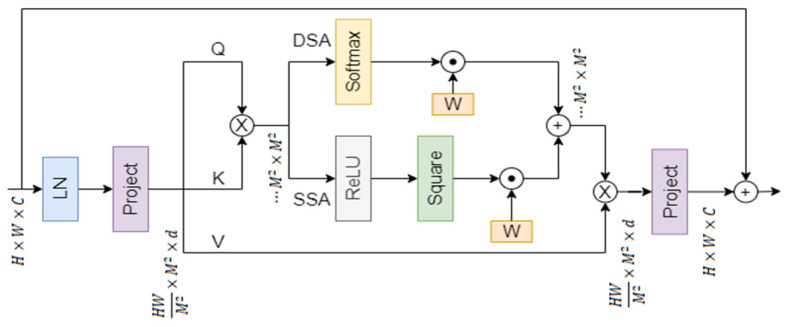
AIFI-ASSA Network Architecture Diagram.

To alleviate the above issue, this study draws on the design concept of Adaptive Sparse Self-Attention (ASSA) in the Adaptive Sparse Transformer [45] and introduces it into the AIFI module of RT-DETR, thereby constructing the AIFI-ASSA adaptive sparse feature interaction module, as shown in Figure 6. While preserving global contextual modeling capability, this module suppresses redundant interactions from low-relevance regions through sparse attention, thereby improving the discriminative representation of defect features under complex orchard backgrounds. Let the input feature be denoted as:

(26) $$X\in R^{C\times H\times W},$$

The input feature is then mapped into the query, key, and value matrices:

(27) $$Q=XW_{Q},$$

(28) $$K=XW_{K},$$

(29) $$V=XW_{V},$$

where $W_{Q}$, $W_{K}\;$ and $W_{V}$ denote the linear projection parameters for the query, key, and value, respectively.

The attention scores are calculated based on the correlation between Q and K:

(30) $$S=\frac{QK^{T}}{\sqrt{d}}+B,$$

where d denotes the feature dimension, B represents the relative positional bias, and S is the attention score matrix. To clarify the dimensions of each tensor, let the spatial size of the input feature map be N=H×W. The query matrix Q, key matrix K, and value matrix V all have dimensions $R^{N\times d}$. Accordingly, the attention score matrix S has dimensions $R^{N\times N}$, and the relative position bias B also has dimensions $R^{N\times N}$. The two are added element-wise and subsequently fed into the sparse and dense attention branches.

In the dense attention branch, the Softmax function is applied to normalize the attention scores, thereby preserving relatively complete contextual information:

(31) DSA=Softmax(S),

Dense attention can sufficiently model global spatial relationships, but it may also introduce redundant interactions from low-relevance background regions. To address this issue, the sparse attention branch employs the squared ReLU function to suppress low-response attention relationships.

(32) $$SSA={ReLU}^{2}(S),$$

where SSA denotes the sparse self-attention branch, which can reduce the influence of low-relevance query–key relationships and encourage the model to focus more on defect-related regions.

To avoid the loss of useful contextual information caused by using sparse attention alone, AIFI-ASSA further introduces learnable weights to adaptively fuse the sparse attention branch and the dense attention branch. Let $a_{1}$ and $a_{2}$ denote the learnable parameters; the weights of the two branches can be expressed as:

(33) $$w_{i}=\sum j=\frac{exp(a_{j})}{\sum_{j=1}^{2}exp(a_{i})},i=1,2,$$

where $w_{1}$ and $w_{2}$ denote the normalized weights of the sparse attention branch and the dense attention branch, respectively.

The final attention output is formulated as:

(34) $$A=(w_{1}\cdot SSA+w_{2}\cdot DSA)V,$$

This dual-branch fusion mechanism can achieve a balance between information preservation and noise suppression. The dense attention branch is used to maintain the necessary global contextual modeling capability, preventing information interactions among important regions from being excessively weakened. In contrast, the sparse attention branch is used to reduce the interference of low-relevance background regions, such as branches and leaves, shadows, specular highlights, and healthy peel textures, during defect feature aggregation.

In the specific implementation, AIFI-ASSA retains the basic structure of the Transformer encoder, including residual connections, normalization operations, and the feed-forward network. The input feature is first processed by AdaptiveSparseSA to perform adaptive sparse feature interaction, and is then added to the original input through a residual connection followed by normalization:

(35) $$X^{\prime }=Norm(X+ASSA(X)),$$

Channel mapping and nonlinear transformation are then performed through a feed-forward network composed of 1 × 1 convolutions:

(36) $$Y=Norm(X^{\prime }+FFN(X^{\prime })),$$

where ASSA(⋅) denotes the adaptive sparse self-attention operation, and FFN(⋅) represents the feed-forward network. In this implementation, the feed-forward network consists of a 1 × 1 convolution, an activation function, and another 1 × 1 convolution, which are used to perform information mapping along the channel dimension.

It should be noted that this study does not fully introduce all components of the Adaptive Sparse Transformer, such as the FRFN module. Instead, only the ASSA attention mechanism is introduced and adapted to the AIFI feature interaction module of RT-DETR. Therefore, the contribution of AIFI-ASSA lies in the adaptive sparsification of the dense feature interaction mechanism in the original AIFI module for citrus defect fruit detection. This design reduces irrelevant token interactions in complex orchard backgrounds and improves the feature representation capability for small-scale defects and weak-texture regions. This module does not claim to re-propose the sparse attention mechanism; rather, it applies the existing adaptive sparse attention concept to optimize high-level feature interaction in agricultural object detection scenarios.

## 3. Results

### 3.1. Dataset and Experimental Setup

**Figure 7:**
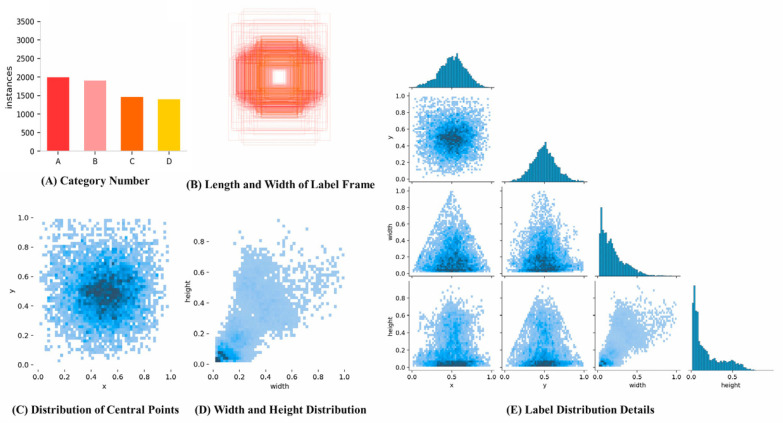
Visualization of the citrus dataset.

The citrus fruit disease image dataset used in this study was primarily sourced from the open Kaggle website, with a small portion collected from the Chu Cheng orange base in Longling County, Baoshan City, Yunnan Province, China (24.25° N, 99.06° E), with an approximate ratio of 6:4. The dataset comprises 1146 images of citrus fruits, categorized into healthy and three common defective states. The image backgrounds include both complex natural environments and simple artificial setups, with natural backgrounds presenting challenges such as leaf and branch occlusion, fruit overlapping, strong light reflection, and backlighting. All images maintain a uniform resolution of 640 × 640 pixels. The dataset includes 338 images of healthy citrus, 323 images of diseased citrus, 248 images of moldy citrus, and 237 images of citrus with severe external damage. To improve the recognition accuracy and generalization ability of the enhanced RT-DETR-DCEA model for citrus defective fruits across different environments, a split-before-augmentation strategy was adopted for the dataset images to avoid data leakage caused by near-duplicate samples entering different data subsets. The images were annotated using the LabelImg tool and divided into training, validation, and test sets with a distribution of 7:2:1, after which data augmentation strategies were applied. These strategies featured horizontal and vertical rotations as well as brightness adjustments, ultimately generating a total of 6756 augmented images. This dataset will be used for experiments including model training, parameter optimization, and prediction result comparison to evaluate model performance. The visualization of the citrus dataset is shown in Figure 7. Figure 7A presents the categories and corresponding label information, where healthy citrus is labeled as A, diseased citrus as B, moldy citrus as C, and citrus with severe external damage as D. Figure 7B displays the dimensions of the label boxes, while Figure 7C illustrates the distribution of center point positions. Figure 7D provides relevant information on the size distribution of citrus fruits, and Figure 7E offers a detailed explanation of the label content. As can be observed, the samples include both large-area defects and localized small lesions and weak-texture targets, and the distribution of target center positions is relatively dispersed, indicating that the model needs to possess both multi-scale feature modeling and complex background suppression capabilities simultaneously.

To ensure the comparability and reproducibility of the experimental results, all experiments in this study were conducted under a unified hardware and software environment. The experimental platform was based on the Windows 11 operating system and was equipped with an AMD Ryzen 9 7945HX processor, an NVIDIA GeForce RTX 4070 GPU, and 32 GB RAM. The programming language was Python 3.10.10, and the deep learning framework was PyTorch 1.13.1. PyCharm was used as the development environment, and the Compute Unified Device Architecture (CUDA) version was 12.0. All comparative models adopted the same dataset partitioning strategy, image input size, and basic training parameters. The input images were uniformly resized to (640 \times 640), the batch size was set to 4, and the number of training epochs was set to 500. The initial learning rate was set to 0.01, and stochastic gradient descent (SGD) was used as the optimizer, with a momentum of 0.937 and a weight decay of 0.0005. During the first three epochs, a warm-up strategy was adopted, in which the learning rate gradually increased from a small value to the initial learning rate to reduce the influence of unstable parameter updates at the early training stage. Subsequently, the default learning rate scheduling strategy of the framework was used to decay the learning rate progressively during training, thereby improving the convergence stability in the later stage. During training and validation, the random seed was fixed at (seed = 0), and deterministic = True was enabled to reduce random errors caused by model initialization, data loading, data augmentation, and the non-determinism of CUDA operators. All ablation and comparative experiments in this study were conducted under this randomness-control setting. The results reported in the tables correspond to a single training run under a fixed dataset split and a fixed random seed. This setting improves experimental reproducibility; however, it is not equivalent to statistical validation using multiple random seeds. Therefore, the need for repeated experiments and uncertainty analysis is further discussed as a limitation and a direction for future work. No modifications or optimizations are made to the loss function in this work. The loss function, as well as the associated weights and other parameters, remains identical to that of the original RT-DETR.

Furthermore, this paper configures the post-processing pipeline according to the original inference paradigm of each detection framework. RT-DETR is an end-to-end real-time detection framework, and one of its core advantages lies in reducing the reliance on Non-Maximum Suppression (NMS) through the DETR-style set prediction mechanism, thereby avoiding the negative impact of NMS post-processing on real-time detection speed and stability. The original RT-DETR paper explicitly states that, as a real-time end-to-end detector, it can eliminate the detrimental effects of NMS post-processing on real-time object detection. Therefore, when evaluating RT-DETR and its improved variants, this paper preserves their end-to-end inference paradigm without additionally introducing conventional NMS post-processing. For the YOLO-series comparison models, including YOLOv8m, YOLOv11m, and YOLOv12m, the default Ultralytics inference settings are uniformly adopted: the confidence threshold is set to 0.25, the IoU threshold to 0.70, and the input size to 640 × 640. Specifically, the confidence threshold is used to filter out low-confidence predictions, and the IoU threshold is applied in the NMS stage to remove highly overlapping redundant bounding boxes. These settings are consistent with the official Ultralytics default inference configuration, which helps minimize the influence of manual parameter tuning on the comparison results across different models. It should be noted that the post-processing mechanisms of RT-DETR and the YOLO-series models are different. Therefore, in the comparative experiments, this paper maintains the original inference paradigm of each model while unifying the data split, input size, training configuration, and evaluation metrics, ensuring that the comparison results are both consistent with each model’s design logic and experimentally comparable.

### 3.2. Model Evaluation Metrics

This paper selects precision (P), recall (R), average precision (AP), mean average precision (mAP), model parameters, and frames per second (FPS) as core evaluation metrics to comprehensively assess the detection performance and operational efficiency of the model. Precision (P) is defined as the ratio of correctly predicted positive samples to all samples predicted as positive, reflecting the accuracy of prediction results; recall (R) is the ratio of correctly identified positive samples to the total actual positive samples, demonstrating the model’s capability to capture positive samples. As key metrics for evaluating object detection performance, mAP@50 uses an Intersection over Union (IoU) threshold of 0.50, whereas mAP@50:95 averages AP over IoU thresholds from 0.50 to 0.95. Therefore, mAP@50 reflects detection performance under a relatively lenient localization criterion, while mAP@50:95 provides a stricter evaluation of localization robustness. The specific calculation is shown in the following formula.

(37) $$P=\frac{T_{P}}{T_{P}+F_{P}},$$

(38) $$R=\frac{T_{P}}{T_{P}+F_{N}},$$

(39) $$AP=\int_{0}^{1}P\left(R\right)dR,$$

(40) $$mAP=\frac{\sum_{i=1}^{C}AP(i)}{C},$$

### 3.3. Model Performance Analysis and Ablation Study

**Figure 8:**
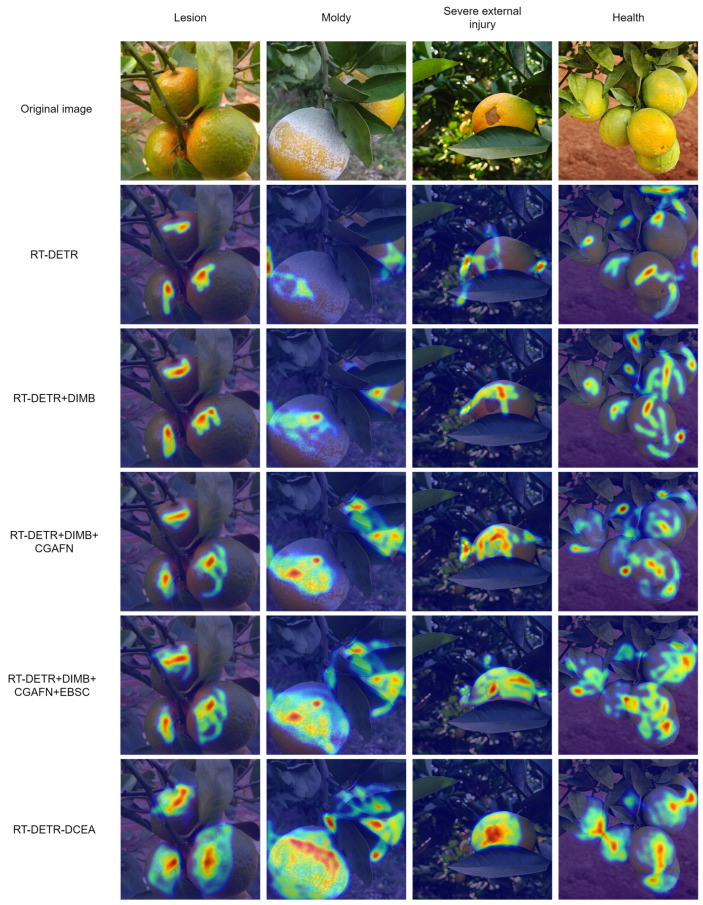
Comparison of heatmaps from different improved models.

**Figure 9:**
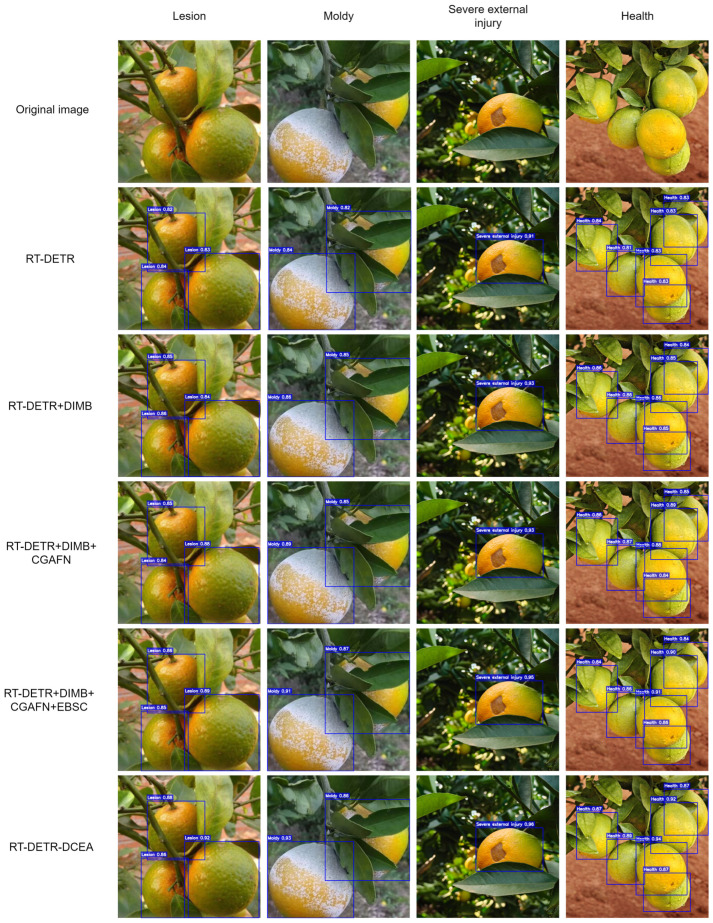
Comparison of effects before and after RT-DETR improvements.

Based on in-depth optimization and improvement of the RT-DETR model, this paper proposes an enhanced model. The expanded dataset is divided into training, testing, and validation sets for training, with analysis conducted on the results of each improvement. The Grad CAM heatmap results of various enhancement modules are shown in Figure 8, the comparison of effects before and after improvement is illustrated in Figure 9, and the ablation experiments of the improved model are presented in Table 1.

The ablation experiments demonstrate that each proposed module improves the detection performance of the baseline model from different perspectives. When DIMB was added alone, Precision increased from 85.7% to 87.8%, indicating that the dynamic multi-morphological convolution enhances the model’s ability to discriminate defect textures and irregular edges. The individual addition of CGAFN raised Recall to 81.2%, suggesting that the content-guided cross-scale feature fusion helps strengthen the complementary relationship between low-level details and high-level semantics, thereby reducing missed detections. Under the single-module setting, EUCB-SC achieved an mAP@50 of 88.8% and an FPS of 91, demonstrating that this module can improve the detail representation of upsampled features with low computational overhead. AIFI-ASSA improved both Recall and FPS, indicating that the sparse-dense attention fusion contributes to suppressing irrelevant feature interactions in complex backgrounds and enhancing the efficiency of information aggregation in high-level features. When DIMB, CGAFN, and EUCB-SC were combined, the model attained an mAP@50 of 91.2%, an improvement of 6.0 percentage points over the baseline. After further incorporating AIFI-ASSA, the final model achieved a Precision of 92.1%, Recall of 86.1%, and mAP@50 of 91.8%. Meanwhile, compared with the baseline model, the final model’s parameter count decreased from 2.008 × 10^7^ to 1.477 × 10^7^, and the FPS increased from 67 to 81, indicating that the proposed improvements achieve accuracy gains not by substantially increasing computational redundancy, but by striking a favorable overall trade-off among detection accuracy, inference speed, and model size. It is noteworthy that the ablation experiment comparing dynamic weighting and static average fusion in DIMB showed that dynamic weighting yielded a 0.7 percentage point improvement in mAP@50 over static averaging, validating the effectiveness of the input-adaptive mechanism (see Table A1 in the Appendix A).

### 3.4. Comparative Experiments on Performance of Different Network Models

To further evaluate the comprehensive performance of the proposed RT-DETR-DCEA model in citrus defective fruit detection tasks, a comparative analysis was conducted with several mainstream object detection models, including YOLOv8m [46], YOLOv11m [47], YOLOv12m [48], and the original RT-DETR model. All comparative experiments were performed under the same dataset, identical training–validation–test splits, and hyperparameter settings to ensure fairness in comparison. The performance comparison is shown in Table 2.

**Figure 10:**
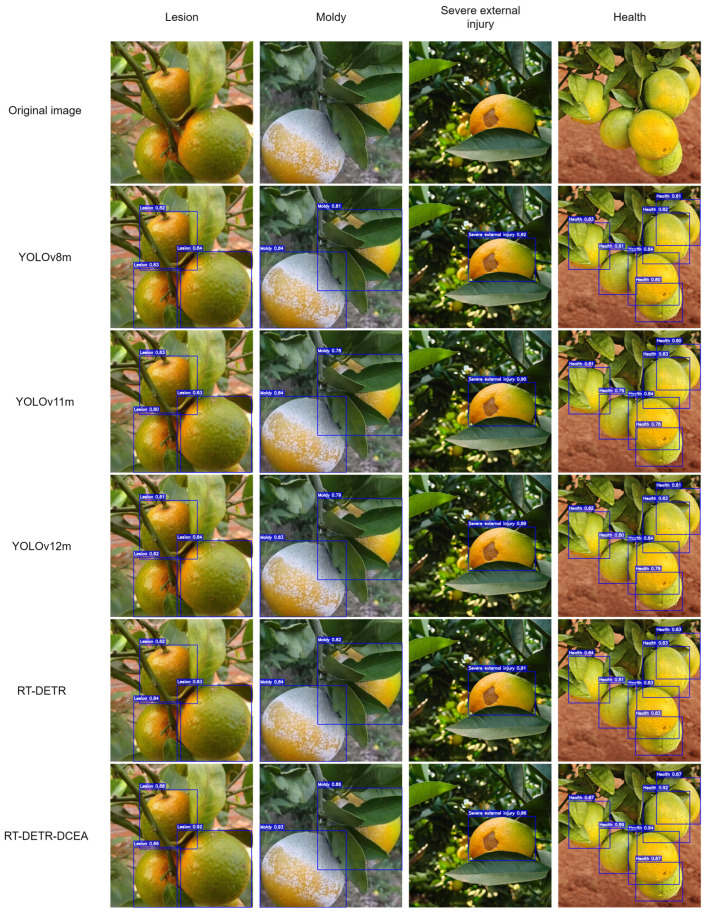
Performance comparison of different network models.

Analysis of the data in Table 2 shows that RT-DETR-DCEA outperforms all compared models on key evaluation metrics, including Precision, Recall, mAP@50, and mAP@50:90, demonstrating the strong overall performance of the proposed method for on-tree citrus fruit recognition and defective fruit detection. Compared with the original RT-DETR-R18, RT-DETR-DCEA achieves improvements of 6.4 percentage points in Precision, 9.4 percentage points in Recall, and 6.6 percentage points in mAP@50, while the number of parameters is further reduced by approximately 26.4% and the FPS increases to 81. These results indicate that the proposed improvements not only enhance detection accuracy, but also improve the lightweight nature and inference efficiency of the model. Compared with YOLOv8m, RT-DETR-DCEA has a slightly lower FPS, yet its Precision, Recall, and mAP@50 are higher by 6.0, 12.3, and 7.4 percentage points, respectively. This suggests that the model possesses stronger recognition capability for small-scale defects, weak-texture lesions, and irregular damage regions in complex orchard scenes, and achieves a favorable balance among detection accuracy, model size, and inference speed. As shown in Figure 10. It should be noted that the current comparative experiments mainly cover YOLO-series models and the original RT-DETR-R18, and have not yet included more strong baselines from the DETR family, such as Deformable DETR, Efficient DETR, or other lightweight RT-DETR variants. Therefore, future studies can incorporate additional DETR-type models under the same data split, training epochs, and evaluation scripts to further validate the relative advantages and generalization performance of RT-DETR-DCEA within the end-to-end detection framework.

**Figure 11:**
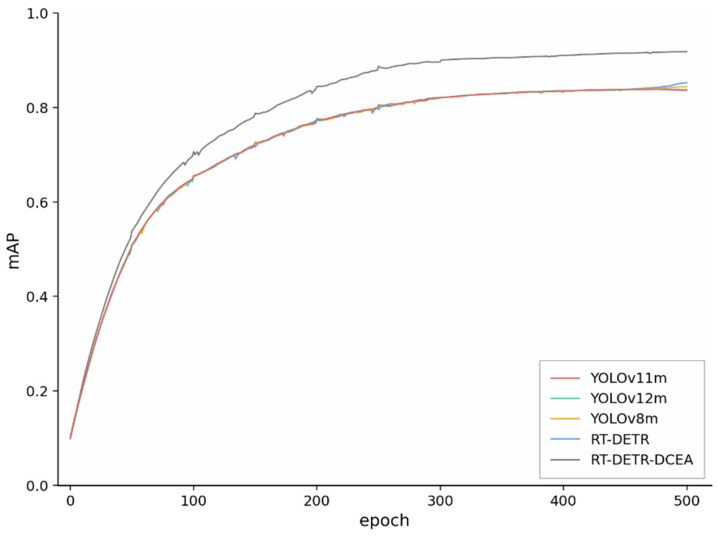
Comparison curves of different models.

**Figure 12:**
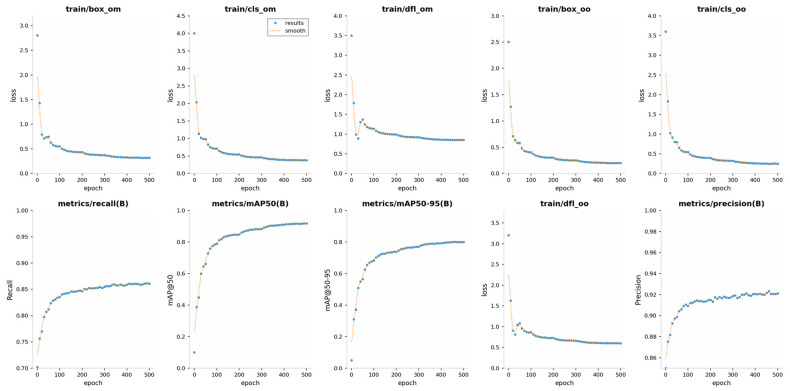
Loss curves.

Figure 11 and Figure 12 shows the changes in training-process metrics for each model under a fixed random seed of (seed = 0) and the same training configuration. It can be observed that the mAP curve of RT-DETR-DCEA remains at a relatively high level in the later training stage, with comparatively small overall fluctuations, indicating that the model exhibits a stable convergence trend under the current dataset split and training settings. Compared with some comparison models, RT-DETR-DCEA achieves higher detection performance in the later training stage, which is generally consistent with the test results reported in Table 2. This suggests that the proposed structural improvements contribute to improved detection performance on the current test set. It should be noted that Figure 11 only reflects the training convergence process under a fixed random seed and the current dataset split. It cannot independently demonstrate the model’s generalization capability across regions, seasons, or scenarios. The statistical stability and external generalization ability of the model still need to be further evaluated through repeated experiments with multiple random seeds, external test sets, and cross-regional data validation.

## 4. Discussion

The performance improvement of RT-DETR-DCEA stems from the synergistic effects of four enhancement modules: DIMB strengthens the backbone’s local representation of multi-morphological defect textures, CGAFN improves the adaptive fusion of cross-scale features in the neck, EUCB-SC enhances detail recovery in the upsampling stage with low parameter overhead, and AIFI-ASSA reduces background noise interference in high-level feature interaction. These four modules target distinct bottlenecks in feature extraction, fusion, recovery, and interaction, respectively, and together yield complementary gains.

From the perspective of computational complexity, the proposed modules improve accuracy without imposing a significant additional computational burden. For an input feature map of size C×H×W, the input-adaptive dynamic weight generation in DIMB introduces only about 3C extra parameters and approximately 3·C·H·W extra FLOPs, the multi-attention mechanism in CGAFN adds about $4C^{2}$ parameters and 8·C·H·W FLOPs, the Shift Channel Mix operation in EUCB-SC introduces no learnable parameters and only incurs roughly 2·C·H·W shift computations, and the dual-branch attention fusion in AIFI-ASSA adds about $2C^{2}$ parameters and 4·N·d FLOPs (where N=H·W). Overall, RT-DETR-DCEA incurs approximately +12% additional FLOPs compared to the original RT-DETR-R18, while reducing the number of parameters by 26.4% (from $2.008\times 10^{7}$ to $1.477\times 10^{7}$). This indicates that the improved performance is not obtained by substantially increasing computational redundancy but rather through precise structural optimization that achieves a favorable balance between accuracy and efficiency.

To quantitatively verify the effectiveness of the dynamic weighting mechanism in DIMB over static average fusion, we conducted a supplementary ablation experiment: the adaptive weights in DynamicInceptionDWConv2d were replaced with equal-weight average fusion $Y=(F_{s}+F_{h}+F_{v})/3$, while all other configurations were kept strictly identical. The results show that the static average fusion achieved an mAP@50 of 84.9%, whereas the proposed dynamic weight fusion reached 85.6%, yielding an absolute improvement of 0.7 percentage points, with negligible additional overhead in parameter count (1.568M vs. 1.567M) and inference speed (79 FPS vs. 80 FPS). This finding confirms that the strong assumption implicit in static averaging—that “features of different morphological defects are equally important”—cannot adapt to the morphological diversity of citrus defects. By generating adaptive weights through global context, DIMB enables the square, horizontal strip, and vertical strip branches to dynamically modulate their contributions according to the local morphology of defects in the input image, thereby more accurately capturing fine-grained differences such as spot-like mold and directional cracks. This ablation experiment provides quantitative support for the input-adaptive mechanism of DIMB and aligns with the aforementioned complexity analysis—achieving a meaningful accuracy gain at a very low additional cost.

**Figure 13:**
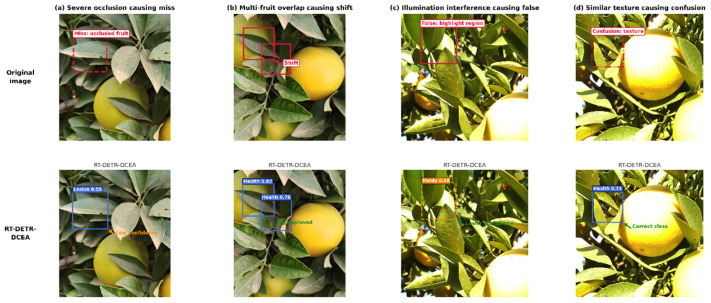
Failure case analysis figure.

Although the overall detection performance is satisfactory, several typical failure modes persist in complex orchard environments. The causes of these failures can be analyzed at both the image feature level and the model mechanism level. As shown in Figure 13, First, false positives induced by illumination interference. Strong light reflection areas and light-colored mold spots exhibit substantial feature overlap in both color and brightness spaces, with closely distributed RGB means and luminance gradients, making it difficult for the model to distinguish them based solely on color and brightness cues, thereby misclassifying highlight regions as defects. Second, false negatives caused by severe occlusion. When foliage occlusion is heavy or only a small portion of the defect is visible, the proportion of valid defect pixels is insufficient, and the local texture features extracted by the backbone become too sparse to support adequate responses during subsequent cross-scale fusion and attention interaction, leading to missed detections. Third, category confusion resulting from texture similarity. The natural texture of healthy fruit skin and the boundary of early-stage lesions exhibit similar local gradient distributions in regions with gradual color changes. Particularly at the initial stage of a lesion, their edge magnitudes and orientation histograms are highly alike, which tends to cause misclassification. Fourth, localization shift due to overlapping fruits. In scenarios with multiple overlapping fruits, where fruit boundaries occlude each other or the background color resembles the skin color, edge responses of different fruits interfere spatially, making it difficult for the bounding box regression branch to accurately delineate object boundaries and thus producing slight offsets. The above phenomena indicate that citrus defect detection performance is influenced not only by model architecture but also jointly by multiple factors such as illumination conditions, occlusion severity, defect development stage, skin texture variation, and background complexity. In essence, they reflect a considerable “domain gap” between image features and defect semantics, which cannot be fully bridged by network structure improvements alone. A coordinated approach encompassing data augmentation, feature learning, and post-processing strategies is required. To address these issues, future work may introduce illumination-invariant feature extraction, occlusion-aware training strategies, and boundary refinement modules during the training phase.

This study has the following limitations. First, the dataset contains only 1146 original images, representing a limited data scale. Data augmentation cannot be equated with independently collected large-scale real samples, and the model’s generalization ability needs to be validated across more regions, cultivars, and seasonal conditions. Second, part of the self-collected data has not been made publicly available due to licensing restrictions of the collection sites, which hinders full reproducibility by third parties. Third, the current experiments are based on a fixed data split and a single training run; the mean, standard deviation, and confidence intervals over multiple random seeds have not been systematically reported. Fourth, on-device latency and power consumption tests have not yet been conducted on edge platforms such as Jetson or Ascend, and the model’s edge deployment performance awaits verification. Fifth, the comparative experiments have not sufficiently included additional strong baselines from the DETR family, such as Deformable DETR and Efficient DETR; the comparison scope should be expanded in the future.

Future research will proceed along the following directions. First, construct a larger and more diverse dataset of citrus defective fruit, covering multiple production regions, cultivars, maturity stages, and illumination conditions. Second, improve the experimental evaluation framework by adding multi-seed repeated experiments, per-class AP, mAP@75, precision–recall curves, confusion matrices, and hard-sample analysis. Third, subject to obtaining full data authorization, further provide an image-based analysis of typical failure cases with geographical annotations to support more transparent model robustness assessment. Fourth, combine deployment techniques such as pruning, knowledge distillation, quantization, and TensorRT acceleration to deploy the model on orchard inspection platforms, edge terminals, or automated sorting lines, and evaluate detection stability, inference latency, and power consumption under long-term operating conditions [49,50,51].

## 5. Conclusions

This paper proposes a lightweight detection model, RT-DETR-DCEA, based on RT-DETR-R18, for on-tree citrus fruit recognition and defective fruit detection in complex orchard environments. To address the challenges of citrus defects, such as small scales, weak boundaries, fine and fragmented textures, large morphological variations, and strong background interference, the original RT-DETR-R18 is improved from four aspects: backbone feature extraction, neck feature fusion, upsampling feature enhancement, and global feature interaction. Specifically, the Dynamic Mixed Convolution Block (DIMB) is introduced to enhance the modeling of local defect textures and directional edges, the Content-Guided Attention Feature Fusion Network (CGAFN) is employed to improve the adaptive fusion between low-level details and high-level semantics, the Efficient Upsampling Block with Shift Channel Mix (EUCB-SC) is designed to strengthen the recovery of fine defect details during upsampling, and the AIFI-ASSA (Adaptive Sparse Feature Interaction) module is utilized to reduce the interference of irrelevant background noise in defect feature representation during high-level feature interaction.

Experimental results on a dataset comprising four categories—healthy, diseased, moldy, and severely externally damaged fruit—show that RT-DETR-DCEA achieves favorable detection performance. The final model attains a Precision of 92.1%, Recall of 86.1%, and mAP@50 of 91.8%, with 1.477 × 10^7^ parameters and an FPS of 81. Compared with the original RT-DETR-R18, RT-DETR-DCEA improves Precision by 6.4 percentage points, Recall by 9.4 percentage points, and mAP@50 by 6.6 percentage points, while reducing the parameter count by approximately 26.4% and further increasing inference speed. Compared with competing models such as YOLOv8m, YOLOv11m, and YOLOv12m, the proposed method demonstrates better detection accuracy and recall capability on the current dataset, indicating that it can more effectively identify small-scale lesions, weak-texture moldy spots, and irregular damage regions in complex orchard scenes. Ablation studies validate the effectiveness of each module, confirming that the performance improvement from combining multiple modules originates from their synergistic optimization across different stages.

It should be noted that the conclusions of this paper are primarily based on the current citrus defective fruit dataset and a desktop GPU experimental platform. Due to the still limited number of original samples and the fact that part of the data was collected in self-built scenarios, the model’s generalization ability across regions, seasons, cultivars, and different acquisition devices requires further validation. Moreover, although the parameter count and FPS indicate a certain potential for real-time detection, systematic on-device tests for latency, power consumption, and long-term stability on actual edge devices have not yet been completed. Therefore, RT-DETR-DCEA currently serves as a feasible lightweight solution for real-time citrus defect detection in complex orchard environments, providing a model foundation for subsequent intelligent orchard inspection, pre-harvest defect screening, and automated grading systems. Future research will further integrate cross-domain data augmentation, model compression, and edge deployment optimization to promote the application of this method in practical agricultural production scenarios.

## Figures and Tables

**Table 1:** Ablation study.

DIMB	CGAFN	EUCB-SC	AIFI-ASSA	P (%)	R (%)	mAP@50 (%)	Params (10^7^)	FPS (Frame/s)
×	×	×	×	85.7	76.7	85.2	2.008	67
√	×	×	×	87.8	78.9	85.6	1.568	79
×	√	×	×	87.2	81.2	87.9	1.989	85
×	×	√	×	88.6	83.8	88.8	1.921	91
×	×	×	√	87.4	81	86.5	2.002	89
√	√	×	×	90.3	84.4	88.6	1.563	82
√	×	√	×	91.1	83	89	1.514	90
√	×	×	√	89.8	84.7	88.9	1.587	87
√	√	√	×	91.7	85.6	91.2	1.489	84
√	√	√	√	92.1	86.1	91.8	1.477	81

Note: √, uses this module; ×, does not use this module.

**Table 2:** Comparison results of different network models in citrus disease detection.

Model	P (%)	R (%)	mAP@50 (%)	mAP@50:95 (%)	Params (10^7^)	FPS (Frame/s)
YOLOv8m	86.1	73.8	84.4	71.8	2.590	84
YOLOv11m	85.1	74.5	83.6	74.7	2.011	82
YOLOv12m	84.7	74.9	83.8	73.6	2.020	66
RT-DETR	85.7	76.7	85.2	74.8	2.008	67
RT-DETR-DCEA	92.1	86.1	91.8	80.1	1.477	81

## Data Availability

The data presented in this study are available on request from the corresponding author due to the data that support the findings of this study are available from the corresponding author upon reasonable request. The data consist of publicly available image resources and data collected by the research team. Publicly available data can be obtained according to the requirements of the original platform. However, the self-collected data are not publicly available due to restrictions on collection site permissions and data usage.

## Appendix A

To verify the effectiveness of the dynamic weighting mechanism in the DIMB module, we replaced the adaptive weights in DynamicInceptionDWConv2d with static average fusion (i.e., forcing $\alpha_{s}=\alpha_{h}=\alpha_{v}=1/3$) under the same experimental settings, while keeping all other structures unchanged. The results are presented in the table below.

**Table A1:** Ablation comparison between dynamic weighting and static average fusion.

Integration Method	P (%)	R (%)	mAP@50 (%)	Params (×10^7^)	FPS
Static average fusion $Y=(F_{s}+F_{h}+F_{v})/3$	86.5	77.2	84.9	1.567	80
Dynamic Weight Fusion $Y=\alpha_{s}F_{s}+\alpha_{h}F_{h}+\alpha_{v}F_{v}$	87.8	78.9	85.6	1.568	79
